# A Multicenter, Randomized Clinical Trial Comparing the Three-Weekly Docetaxel Regimen plus Prednisone versus Mitoxantone plus Prednisone for Chinese Patients with Metastatic Castration Refractory Prostate Cancer

**DOI:** 10.1371/journal.pone.0117002

**Published:** 2015-01-27

**Authors:** Tie Zhou, Shu-xiong Zeng, Ding-wei Ye, Qiang Wei, Xu Zhang, Yi-ran Huang, Zhang-qun Ye, Yong Yang, Wei Zhang, Ye Tian, Fang-jian Zhou, Jin Jie, Shi-ping Chen, Yan Sun, Li-ping Xie, Xing Yao, Yan-qun Na, Ying-hao Sun

**Affiliations:** 1 Department of Urology, Changhai Hospital, The Second Military Medical University, 168 Changhai Road, Shanghai 200433, P.R. China; 2 Department of Urology, Shanghai Cancer Center, Fudan University, Shanghai, P.R. China; 3 Department of Urology, West China Hospital, West China School of Medicine, Sichuan University, Chengdu, P.R. China; 4 Department of Urology, Chinese PLA General Hospital, Medical School of Chinese PLA, Beijing, P.R. China; 5 Department of Urology, Renji Hospital, Shanghai Jiaotong University School of Medicine, Shanghai, P.R. China; 6 Department of Urology, Tongji Hospital, Tongji Medical College, Huazhong University of Science & Technology, Wuhan, P.R. China; 7 Department of Urology, Beijing Cancer Hospital, Peking University, Beijing, P.R. China; 8 Department of Urology, Jiangsu Province Hospital, Nanjing Medical University, Nanjing, P.R. China; 9 Department of Urology, Beijing Friendship Hospital, Capital Medical University, Beijing, P.R. China; 10 Department of Urology, Cancer Hospital, Sun Yat-Sen University, Guangzhou, P.R. China; 11 Department of Urology, Peking University First Hospital, Beijing, P.R. China; 12 Department of Urology, Fujian Union Hospital, Fuzhou, P.R. China; 13 Department of Urology, Cancer Hospital, Chinese Academy of Medical Sciences, Beijing, P.R. China; 14 Department of Urology, the First Affiliated Hospital, Zhejiang University, Hangzhou, P.R. China; 15 Department of Urology, Tianjin Cancer Hospital, Tianjin Medical University, Tianjin, P.R. China; 16 Department of Urology, Shougang Hospital, Peking University, Beijing, P.R. China; Wayne State University School of Medicine, UNITED STATES

## Abstract

**Purpose:**

To explore the feasibility and efficacy of docetaxel plus prednisone for Chinese population with metastatic castration refractory prostate cancer (mCRPC).

**Patients and methods:**

A total of 228 patients recruited from 15 centers were randomized to receive 10 cycles of D3P arm (docetaxel: 75 mg/m^2^, intravenous infusion, every three weeks; Prednisone 10mg orally given daily) or M3P arm (mitoxantrone: 12 mg/m^2^, intravenous infusion, every three weeks; Prednisone 10mg orally given daily). Primary end point was overall survival, and secondary end points were events progression-free survival (PFS), response rate, response duration. Quality of life (QoL) was also assessed in both treatment groups.

**Results:**

The median overall survival was 21.88 months in D3P arm and 13.67 months in M3P arm (P = 0.0011, hazard ratio = 0.63, 95% confidence interval, 0.46–0.86). Subgroup analysis was consistent with the results of overall analysis. Events progression-free survival (pain, PSA, tumor and disease) were significantly improved in D3P arm compared with M3P arm. PSA response rate was 35.11% for patients treated by D3P arm and 19.39% for M3P arm (P = 0.0155). Pain response rate was higher in D3P arm (61.11%, *P* = 0.0011) than in M3P (23.08%) arm. No statistical differences were found between D3P arm and M3P arm for QoL, tumor response rate and response duration of PSA and pain. The tolerability and overall safety of D3P arm were generally comparable to that of M3P arm.

**Conclusions:**

Compared with M3P arm, D3P arm significantly prolonged overall survival for the Chinese patients with mCRPC and improved the response rate for PSA and pain.

**Trial Registration:**

clinicaltrials.gov NCT00436839

## Introduction

Prostate cancer is one of the most common cancers in elderly men, a total of 233,000 new prostate cancer cases are projected to occur in the USA in 2014. [1] Meanwhile, due to life-style change, frequent PSA monitor, advanced radiological investigations and enlarged elderly population, prostate cancer has become one of the fastest growing cancers in China. The incidence of prostate cancer in Shanghai has increased by almost 4.8 folds in the last 27 years and become the most common genitourinary tumor in males in this geographic area. [2]

Once prostate cancer progresses to metastasis castration refractory stage (mCRPC), the prognosis is dismal with a median survival time of 9 to 13 months. [3] Mitoxantrone and prednisone were once considered the standard of care for mCRPC patients. However no study found this approach could improve overall survival. [4, 5] In 2004 two landmark studies TAX-327 [6] and SWOG-9916 [7] unprecedentedly proved that docetaxel based chemotherapy could prolong overall survival (OS) and improve response rate of pain, serum prostate specific antigen (PSA) and quality of life (QoL) for mCRPC patients, which led to the approval of docetaxel as the first line chemotherapy for mCRPC by the USA Food and Drug Administration (FDA) in 2004 and European Medicines Agency in 2005. [8] The latest update data of TAX-327 trial showed that the median overall survival time was 19.2 months in the docetaxel plus prednisone arm and 16.3 months in the mitoxantrone plus prednisone arm (Hazard ratio = 0.79, 95% confidence interval = 0.67 to 0.93, P = 0.004). [9]

Whether docetaxel-based chemotherapy will benefit the Asian population is still controversial, because of the lack of strong support from clinical trials. [10–13] The clinical data of TAX-327 and SWOG-9916 were collected from major clinical centers around the world but without Asia centers. [6, 7] What’s more, no phase III clinic trial of docetaxel-based chemotherapy has been conducted among Chinese population. In order to explore the feasibility and efficacy of docetaxel treatment for mCRPC patients among Chinese population, we conducted this prospective, randomized, parallel controlled, open-label, multicenter phase III study.

## Materials and Methods

The protocol for this trial and supporting CONSORT checklist are available as supporting information; see S1 Protocol and S1 CONSORT Checklist.

### Selection of study population

The clinical trial regimen, with a study code **NCT00436839**, was based on the pivotal trial TAX-327. [6] The authors confirmed that all ongoing and related trials for this drug were registered. The registration of this study was processed by the data transparency team of Sanofi Global. All data to be disclosed needed to go through an internal review & approval process before final releasing. The review process was time-consuming, which caused first patient enrolled occurred before trial registration date. Patients were enrolled in this trial from 15 investigative centers in China. Patients were included in the study according to the following criteria. The inclusion criteria included: histologically or cytologically confirmed prostate adenocarcinoma, Karnofsky performance status (KPS) ≥70, life expectancy≥3 months, documented metastatic prostate cancer that was unresponsive or refractory to hormone therapy, documented progression detected by PSA increase (two consecutive rises in PSA taken at least one week apart, a fourth PSA measure would be required if the third one was not greater than the second PSA measure), prior surgical therapy and radiotherapy (with bone marrow depression risk ≤25%) was allowed (at least 4 weeks must had elapsed since completion of these therapies and recovered from side effects), prior treatment with corticosteroids was allowed, effective androgen deprivation by surgical castration (orchiectomy) and/or drug castration (If the patient had been treated with LHRH agonists without orchiectomy, this therapy should be continued) were needed (testosterone level≤50ng/dL), compliance with the anti-androgen withdrawal period (i.e. flutamide, nilutamide, cyproterone acetate had stopped at least 4 weeks, bicalutamide had stopped at least 6 weeks), achieved stable analgesia (i.e. an increase≤1 point in daily present pain intensity (PPI) scale and analgesic scores (AS) variation within ±25% of the mean AS) for a minimum of 7 consecutive days prior to randomization.

Exclusion criteria: prior chemotherapy (with the exception of estramustine and a wash-out period of at least 4 weeks), isotope therapy within the last 3 months, prior malignancy (excluding adequately treated basal cell skin cancer, any other cancer with disease-free for more than 5 years), brain or leptomeningeal involvement, symptomatic peripheral neuropathy ≥ grade 2 (National Cancer Institute-Common Toxicity Criteria, NCI-CTC, version3), concurrent treatment with other investigational drugs, other serious medical condition (e.g. congestive heart failure, peptic ulcer), abnormal laboratory examination (i.e. neutrophils < 1.5×10^9^/L, hemoglobin < 10g/dl, platelets < 100×10^9^/L, total bilirubin > the upper limit of normal range, creatinine, ALT and AST > 1.5 times the upper limit of normal range).

All patients provided their written informed consent, and the protocol complied with recommendations of the 18th World Health Congress and all applicable amendments. The protocol also complied with the laws and regulations, as well as any applicable guidelines of China where the study was conducted. All the below ethic committees specifically approved this study: Shanghai Changhai hospital; Cancer Hospital, Chinese Academy of Medical Sciences; Tongji Hospital, Wuhan Tongji Medical University; Beijing Cancer Hospital, Peking University; Chinese PLA General Hospital, Medical School of Chinese PLA; Tianjin Cancer Hospital, Tianjin Medical University. The first approval of the study by the ethic committee was at 18^th^, October in 2006.

### Outcome measures

Primary outcome was OS, secondary outcomes were events (e.g. PSA, pain, tumor, disease) progression-free survival (PFS), response rate, response duration and QoL. The definition and criteria for primary and secondary outcome were listed in S1 Table.

### Treatment plan

D3P arm: docetaxel (Taxotere; Sanofi Aventis) was given intravenous at 75mg/m^2^, day 1 every three weeks plus prednisone 10mg orally given daily. M3P arm: mitoxantrone was given intravenous at 12mg/m^2^, day 1 every three weeks plus prednisone 10 mg orally given daily. Patients were randomly assigned to receive either D3P arm or M3P arm by centralized permuted-block randomization, and stratified by pain level (PPI≥2 vs. <2) and performance status (KPS≥80 vs. <80). Treatments for both regimens were planned for 10 cycles. If the calculated body surface area (BSA) of the patient was>2.0m^2^, the dose to be given to the patient was calculated according to BSA = 2.0m^2^. Premedication with dexamethasone 7.5mg was administered 12 hours, 3 hours, and 1 hour before the docetaxel infusion.

If toxicity occurred, appropriate treatment would be used to ameliorate signs and symptoms. If a patient experienced severe toxicities and there were conflicting treatment recommendations, the most conservative dose adjustment (D3P: level one 60mg/m^2^ and level two 45mg/m^2^, M3P: level one 10 mg/m^2^ and level two 8 mg/m^2^) would be adopted. No more than 2 dose reductions would be implemented per patient. A chemotherapy treatment delay needed to be justified and to be recorded in the case report form if delayed more than 4 days. Normally, treatment could be delayed no more than 2 weeks to allow recovery from acute toxicity. Otherwise, the patient went off the study. Patients received 10 cycles of treatment in D3P arm and M3P arm, unless the following events occurred earlier: unbearable severe adverse effect, progression of disease, withdrawal of consent, administration of any other anti-tumor therapy, or lost to follow up.

### Pretreatment and follow-up evaluations

Complete history; physical examination and radiological imaging were collected within 14 days before the randomization. Blood tests including PSA and hematology, cardiovascular function evaluation, PPI and QoL were also assessed at baseline. History; physical examination; biochemistry; PSA; QoL; adverse effect; PPI were measured routinely every three weeks before drug infusion. Hematology was repeated at least once a week or every 2 days if clinically indicated. Radiology and bone isotope scanning were conducted at weeks 6, 12, 21 and 30 since treatment began. Left ventricular ejection fraction was required in D3P arm only following cycles 5, 8, 10 or clinically indicated. Patients would be followed up after finishing chemotherapy monthly until death.

### Toxicity

Safety data included adverse events, clinical examinations, vital signs, KPS and clinical tests (i.e. hematology and biochemistry, electrocardiograms, LVEF measurement, and chest X-ray). Clinical symptom and laboratory toxicity would be graded according to NCI-CTC toxicity standard (version 3.0), and common hematologic toxicities criteria were listed in S2 Table. Prior to dose administration of every cycle, adverse event, disease symptom/signs, and blood chemistry were assessed. Blood routine test was assessed once a week.

### Statistical methods

The hypothesis of the study was the D3P arm was able to improve 35% of median survival compared with the M3P arm. It was assumed that 3 years were needed for patients recruit and 2 additional years for follow-up. Therefore, it was estimated that a sample size of 88 patients per group were needed, using a one-sided test with a type I error rate of 5% and statistical power of 80%. However, 20% patients were estimated not assessable for primary outcome and at least 100 pairs of valid patients were required to test the efficacy of drugs for a new indication according to the relevant regulations in China. As a result, 228 patients were finally enrolled in this clinical trial. The primary analysis was to compare OS in the intent to treat (ITT) population using Kaplan-Meier curve and log-rank test, stratified by baseline pain (PPI ≥2 vs. <2) and performance status (KPS ≥80 vs. < 80) at randomization. Testing at two-sided 5% significance level was performed. The Kaplan-Meier curve was used to describe survival data in each analysis, hazard ratios (HR) and 95% confidence intervals (CI) were presented.

Secondary efficacy analyses for events progression-free survival in ITT population were conducted using Kaplan-Meier curve and log-rank test for pain, PSA, tumor, and disease. Response rates for pain, PSA, tumor, QoL were calculated in ITT population using chi-square or Fisher’s exact test. Duration of pain and PSA responses were analyzed using log-rank test. Regardless of patients’ eligibility for the study, patients who received at least one infusion of study chemotherapy or one dose of prednisone, were included in the safety set (SS) population. The safety analyses were to compare the safety parameters between the two treatment groups in the safety population.

## Results

### Baseline demographics and characteristics

**Figure 1:**
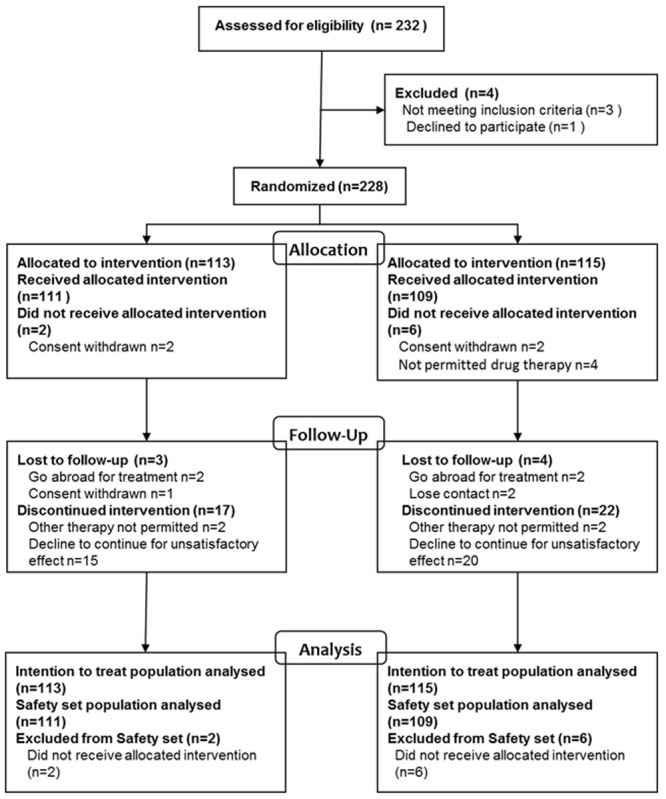
CONSORT diagram.

Between January 2007 and December 2010, a total of 228 patients were randomized at 15 Chinese centers in China; 220 patients were treated and entered into SS population (Fig. 1). The patient baseline characteristics were well balanced between the two groups (Table 1). Of the eligible patients, 44 (38.94%) in the D3P arm and 40 (34.78%) in the M3P arm reported bone pain on study entry (P>0.05).

**Table 1:** Demographics and baseline patient characteristics.

	**D3P**	**M3P**
No. of patients	113	115
Age, years		
Median(Range)	70.7(44.3–82.3)	70.8(49.6–81.1)
≧65**(n%)**	81(71.68%)	84(73.04%)
KPS		
Median (range)	90 (70–100)	90 (60–100)
≥80**(n%)**	102 (90.27%)	96 (83.48%)
PPI		
Median (range)	1 (0–5)	1 (0–4.14)
≥2**(n%)**	32 (28.32%)	29 (25.22%)
Gleason Score		
Median(Range)	8(4–10)	8(6–10)
≥8(**n%**)	50(44.25%)	42(36.52%)
FACT_P		
Median(Range)	110.14(57–148)	105.00(55–139)
Duration of prostate cancer, years		
Median (range)	2.05(0.5–10.14)	1.91(0.35–10.31)
Type of site involved*(n%)		
Bone	97 (85.84%)	98 (85.22%)
Lymph nodes	21 (18.58%)	19 (16.52%)
Visceral	21 (18.58%)	20 (17.39%)
Liver	2 (1.77%)	8 (6.96%)
Lung	11 (9.73%)	9 (7.83%)
Other organs	8 (7.08%)	3 (2.61%)
Other soft tissues	11 (9.73%)	4 (3.48%)
Baseline PSA, ng/ml		
Median (range)	70.90(1.00–2031.00)	100.00(0.06–3387.83)
Prior anticancer therapy **(n%)**		
Prostatectomy	26 (23.01%)	25 (21.74%)
Radiotherapy	91 (80.53%)	87(75.65%)
Hormonal therapy	112 (99.12%)	109 (94.78%)
Orchidectomy	90 (79.65%)	85 (73.91%)
Anti-androgens	108 (95.58%)	106 (92.17%)
Estramustine	97 (85.84%)	100 (86.96%)

**Abbreviations:** D3P = docetaxel every three weeks plus prednisone; M3P = mitoxantrone every three weeks plus prednisone; KPS = Karnofsky performance status; PPI = Present Pain Intensity; FACT_P = Functional Assessment of Cancer Therapy–Prostate; PSA = serum prostate specific antigen.

### Administration of investigational drugs

Treatment distribution and reasons for therapy discontinuation were shown in Table 2. The most common reason for dose reduction was hematologic toxicity in both arms. Of note, 181 (23.03%) and 126 (23.82%) cycles were delayed on D3P and M3P arm respectively, but the majority of reasons for cycles delay were unrelated to adverse effect, e.g. personal problems, logistical issues, or vacation. In fact, only 26 (3.31%) cycles delay in D3P arm and 36 (6.81%) in M3P arm were experimental drug-related. Of those patients not completing 10 cycles, the main reasons for discontinuation was disease progression and death. A higher percentage of patients in M3P group discontinued study due to disease progression or death compared to D3P group (35.65% vs 27.43%, P>0.05).

**Table 2:** Drug delivery and reasons for treatment discontinuation.

	**Treatment group^†^**	
	**D3P**	**M3P**
No. of patients	113	115
No. of patients receiving study medicine	111	109
No. of total cycles received	786	529
Median cycles received by patient	8	4
Median cumulative dose, mg/m^2^	567.18	46.58
No. of patients with dose reduction* (n%)	21(18.92%)	11(10.09%)
One dose reduction	13(11.71%)	9(8.26%)
Two dose reduction	8(7.21%)	2(1.83%)
No. of total cycles delayed^#^ (n%)	181(23.03%)	126(23.82%)
Adverse effect unrelated to study medicine	14(1.78%)	4 (0.76%)
Hematologic toxicity	15(1.91%)	23 (4.35%)
Non-hematologic toxicity	10(1.27%)	10 (1.89%)
Hematologic and non-hematologic toxicity	1(0.13%)	3 (0.57%)
Others	141(17.94%)	86 (16.26%)
Reasons for treatment discontinuation (n%)		
Completed treatment	50(44.25%)	27(23.48%)
Progressive disease or death	33(29.20%)	43(37.39%)
Adverse event	9(7.96%)	19(16.52%)
Lost to follow-up	3(2.65%)	4(3.48%)
Other therapy not permitted	2(1.77%)	2(1.74%)
Consent withdrawn	1(0.88%)	0(0.00%)
Decline to continue further treatment	15(13.27%)	20(17.39%)

^†^ Because of rounding, not all percentages total 100.

* No more than 2 dose reductions would be implemented per patient.

^#^ Treatment could be delayed no more than 2 weeks.

### Treatment efficacy

**Figure 2:**
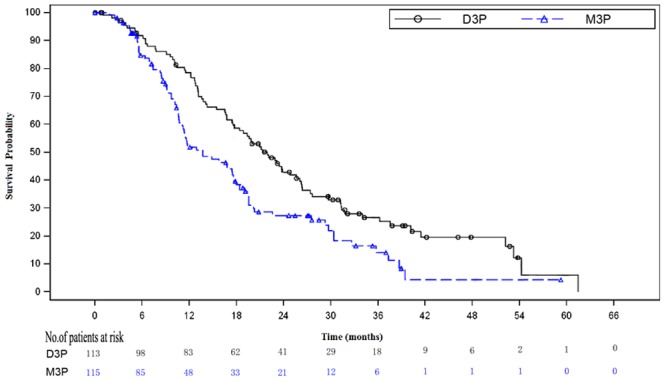
Kaplan-Meier curve for overall survival by different treatment arms in the intention to treat analysis. The Kaplan-Meier curve for D3P arm was above the curve for M3P arm, log-rank analysis proved this difference was statistically significant (P = 0.0011, HR = 0.63, 95% CI [0.46–0.86]). D3P = docetaxel plus prednisone arm. M3P = mitoxantrone plus prednisone arm.

**Figure 3:**
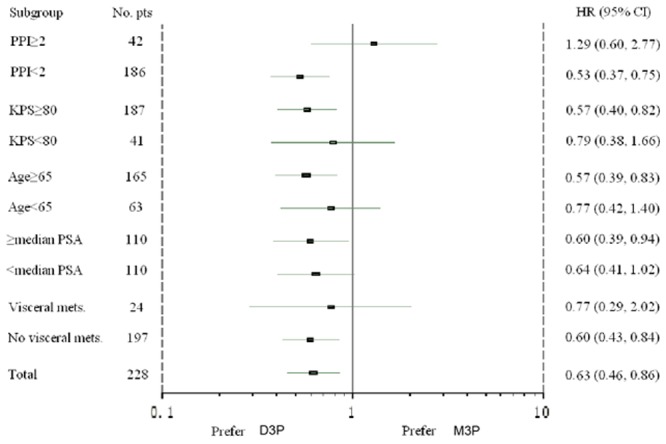
The primary analysis on overall survival in subgroups. Patients in D3P arm and M3P arm were divided into subgroups by stratification variables (baseline PPI and KPS) and prognostic factors of interest (age, baseline PSA and visceral involvement). There were no statistically difference between two arms in subsets of PPI≥2，KPS≥80，median PSA<110ng/ml and visceral metastasis, and subgroup analysis showed favorable results for D3P arm in the rest of subsets. PPI = present pain intensity; KPS = Karnofsky performance status; PSA = prostate specific antigen; visceral mets. = visceral metastasis.

The median follow-up time was 39.13 months for D3P group and 28.45 months for M3P group. The median overall survival was 21.88 months in D3P arm and 13.67 months in M3P arm (P = 0.0011, Fig. 2), the corresponding HR for death was 0.63 (95% CI, 0.46 to 0.86). Primary analysis on OS was repeated in subset groups defined by the stratification variables (baseline PPI and KPS) or the prognostic factors of interest (age, baseline PSA, visceral involved). The results of the subgroup analyses were consistent with the.overall analysis, as shown in Fig. 3.

The results of secondary outcome demonstrated that patients in the D3P arm had significantly prolonged events (i.e. PSA, pain, tumor, disease) PFS compared with M3P arm, as shown in Table 3. Of note, PSA and pain response rate between the two arms were statistically different, but PSA and pain response duration was not statistically significant between the D3P arm and M3P arm, respectively. Furthermore, although tumor response rate and QoL evaluation were in favor of D3P arm, the differences between the two arms were not statistically significant.

**Table 3:** Results of secondary outcome.

	**D3P**	**M3P**	**P value**
PSA Response^†^			
PSA response ITT	94	98	
PSA response rate(95%CI*)	35.11% (25.54%-45.64%)	19.39% (12.10%-28.61%)	0.0155**
PSA response duration, months(95%CI)	4.2(3.48–4.86)	2.83(1.51–4.83)	0.0792
PSA Progression-Free Survival, months Median(95%CI)	12.71(7.65–17.51)	5.55(3.48–8.9)	0.0001
Pain Response^†^			
Pain response ITT	36	39	
Pain response rate(95%CI*)	61.11% (43.46%-76.86%)	23.08% (11.13%-39.33%)	0.0011**
Pain response duration, months(95%CI)	3.71(1.84–4.93)	2.17(0.72–4.99)	0.2309
Pain Progression-Free Survival, months Median(95%CI)	12.71(9.53–15.47)	5.55(3.61–8.44)	0.0002
Tumor Response^†^, (n%)			
ITT for tumor efficacy assessment	29	31	
Complete Response Rate	1(3.45%)	0	
Partial Response Rate	6(20.69%)	5(16.13%)	
no change	10(34.48%)	10(32.26%)	
progressive disease	3(10.34%)	2(6.45%)	
Tumor Response Rate	7(24.14%)	5(16.13%)	0.5269**
Tumor Progression-Free Survival, months Median(95%CI)	12.19(8.05–13.76)	9.13(6.93–10.71)	0.0118
Disease progression-free survival, months Median(95%CI)	3.42(2.79–4.96)	2.14(1.61–2.76)	0.0029
Quality of Life^†^			
Assessment for QoL, ITT	81	76	
Response Rate(95%CI*)	19.75% (11.73%-30.09%)	15.79% (8.43%-25.96%)	0.5393**

**Abbreviations:** CI = confidence interval; ITT = intention to treat; QoL = quality of life.

*Clopper-Person exact method was used to evaluate confidence interval.

**Fisher exact probabilities method was used, 2-sided P-value.

^†^ Definition and criteria of PSA, Pain, Tumor, QoL response were listed in S1 Table.

### Safety results

An overview of treatment-emergent-adverse-events (TEAEs) in safety set analysis was shown in Table 4. The most frequent toxicities were neutropenia (D3P: 58.56%, M3P: 53.21%) and leukopenia (D3P: 19.82%, M3P: 17.12%) in both arms. Although incidence of grade 3–5 neutropenia was relatively high, febrile neutropenia was rare. There were 3 (2.7%) patients in D3P arm and 2 (1.83%) patients in M3P arm died from chemotherapy related toxicity. Eight (7.21%) patients dropped out from D3P arm for TEAEs-related reasons, the most common of which were leukopenia (n = 2, 1.80%) and lung infection (n = 2, 1.80%); Fifteen patients (13.76%) in M3P arm withdrew out of TEAEs-related reasons, the most common of which were neutropenia (n = 3, 2.75%) and infection (n = 2, 1.83%). The analysis of the safety data demonstrated that the tolerability and overall safety of D3P arm was generally comparable to that of M3P arm.

**Table 4:** A summary of the incidence of TEAEs in the safety population.*

**TEAEs**	**Treatment group**			
	**D3P (n = 111)**		**M3P (n = 109)**	
	**All grades (n%)**	**Grade 3–5 (n%)**	**All grades (n%)**	**Grade 3–5 (n%)**
Neutropenia	65 (58.56)	64 (57.66)	58 (53.21)	50 (45.87)
Leukopenia	22 (19.82)	19 (17.12)	33 (30.28)	25 (22.94)
Alopecia	9 (8.11)	4 (3.6)	3 (2.75)	1 (0.92)
Infection	6 (5.41)	5 (4.5)	6 (5.5)	5 (4.59)
Fever	6 (5.41)	4 (3.6)	1 (0.92)	1 (0.92)
Anemia	5 (4.5)	4 (3.6)	4 (3.67)	3 (2.75)
Nausea	5 (4.5)	0 (0.00)	4 (3.67)	1 (0.92)
Fatigue	4 (3.6)	0 (0.00)	10 (9.17)	4 (3.67)
Diarrhea	4 (3.6)	2 (1.8)	0 (0.0)	0 (0.0)
Febrile neutropenia	2 (1.8)	2 (1.8)	0 (0.0)	0 (0.0)
Pharyngitis	2 (1.8)	1 (0.9)	2 (1.83)	0 (0.0)
Oral ulcer	2 (1.8)	1 (0.9)	2 (1.83)	0 (0.0)
Hypokalemia	2 (1.8)	2 (1.8)	1 (0.92)	1 (0.92)
Cardiac failure	1 (0.9)	1 (0.9)	0 (0.0)	0 (0.0)
Peripheral edema	1 (0.9)	0 (0.0)	0 (0.0)	0 (0.0)
Erythra	1 (0.9)	0 (0.0)	0 (0.0)	0 (0.0)
Vomiting	1 (0.9)	0 (0.0)	3 (2.75)	1 (0.92)
Bone pain	1 (0.9)	0 (0.0)	3 (2.75)	2 (1.83)
Hyponatremia	1 (0.9)	1 (0.9)	2 (1.83)	2 (1.83)
Allergic reactions	1 (0.9)	0 (0.0)	0 (0.0)	0 (0.0)
Constipation	1 (0.9)	0 (0.0)	0 (0.0)	0 (0.0)

**Abbreviations:** TEAEs = treatment-emergent-adverse-events；NCI-CTC = National Cancer Institute-Common Toxicity Criteria.

* The sequence of TEAEs was in the order of decreasing frequency, ordered by D3P arm.

## Discussion

Metastatic castration refractory prostate cancer is defined either on the basis of rising PSA, or with evidence of clinical or radiological disease progression despite of castration levels of testosterone. [14] Once prostate cancer reaching this advanced phase, treatment for mCRPC becomes a significant challenge to urologists. [14, 15] Although systemic chemotherapy for mCRPC has been evaluated for many years, prostate cancer had been considered to be a chemo-resistant disease for disappointed earlier clinical trial outcomes. Mitoxantrone had been shown to have benefits in palliating bone pain and improving QoL, but it did not improve overall survival, with the median survival about 10–13 months. [3, 7, 8] Since benefits in overall survival and symptoms palliative were demonstrated by the two cornerstone trials (TAX-327 [6] and SWOG-9916 [7]), docetaxel have become the standard chemotherapy agent for mCRPC patients. It was reported that docetaxel-based chemotherapy could prolong overall survival to 17.5–22.6 months. [15] Although the last four years have witnessed sipuleucel-T, cabazitaxel, abiraterone acetate, enzalutamide and radium-223 been approved by FDA as effective treatment for CRPC patients because of their advantages in prolonging overall survival, docetaxel-based therapy remains the first-line and most widely accepted treatment for mCRPC. [16]

In the present study, the median survival of D3P arm was 21.88 months, which was similar to the result reported by Kelly et al.(22.6 months) [17]and a bit longer than the result reported by Berthold et al.(19.2 months). [9] The PSA response rate of D3P arm in previous phase III clinical trial ranged from 45% to 58%. [6, 17] In spite of a satisfactory overall survival in D3P arm, it was noteworthy that the PSA response rate (35.11%) in D3P arm of the present study was lower than the previous results. Although Zhang et al. [13] reported that the PSA response rate was 43.2% in D3P arm based on a Chinese population, this study was a non-randomized clinical trial based on only 44 patients in D3P arm. The discrepancy between PSA response rate and overall survival might due in part to that PSA response rate might not parallel with the OS. [18, 19] Furthermore, the Flare phenomenon of PSA after initial therapy was another possible reason. It was advised that recognizing a favorable effect on PSA should be delayed for 12 weeks or more for cytotoxic drugs, and treatment plan should be beyond early PSA rises unless other evidence of progression occurred. [18–20] However in the present study the Flare phenomenon was not taken into account since the trial start earlier than this phenomenon put forward in 2008 by Prostate Cancer Clinical Trials Working Group 2 (PCWG2)[19].

Contradictory to the present study and previous results, the study conducted by Abu-Hamar [10] showed that median OS was 15 months for mCRPC patients with D3P arm, and they speculated that the docetaxel-based therapy presented a poor survival outcome for Egyptian. Meanwhile, Thatai et al. [21] reported that the time to PSA progression was significantly longer for whites than for blacks, while no statistically significant difference by race was found in OS and PSA response rate. Recently, pharmacogenetic studies have identified distinct ethnic differences in genetic polymorphisms that were potentially involved in efficacies and toxicities of anticancer drugs. [22, 23] However, there was little comparative data regarding the response and toxicity of chemotherapy regimens for mCRPC among different racial groups. Up until now, there was no phase III docetaxel-based chemotherapy clinical trial for mCRPC patients in Asia. To our knowledge, this study was the first report introducing corresponding data.

This trial identified docetaxel was as tolerable as mitoxantrone, which was consistent with the reports of other studies. [6, 7, 9, 13, 24] Compared with M3P arm (89.91%), TEAEs happened more frequently in D3P arm (94.59%). Nevertheless, D3P arm led to less withdrawal (8.11%) than M3P arm (13.76%). Most of the adverse events observed were hematological toxicity, among which neutropenia was the most common adverse effect. The incidence of grade 3/4 neutropenia of the D3P arm of the present study was similar to that reported by Naito et al. (93%) [8] and Abu-Hamar et al.(85.7%), [10] while much higher than TAX-327 (32%). [6] Febrile neutropenia (5.41%) of the D3P arm in this study was close to TAX-327 (3%), nevertheless lower than the results of Naito et al.(16.3%) [8] and Abu-Hamar et al.(14.3%). [10] It was likely that population ethnicity might have an impact on both appropriate treatment dosage and response. For instance, Naito et al. [8] adopted a 70 mg/m^2^ dose for D3P arm, which was considered more appropriate for Japanese patients based on their studies of maximum tolerated dose of docetaxel.

Several intrinsic limitations of this study could not be neglected. To begin with, the baseline characteristics were well balanced except that the PSA level of M3P arm (100ng/ml) was higher than D3P arm (70.9ng/ml). It was accepted that PSA reflect tumor burdens, the lower PSA level of D3P arm may cause favorable bias for it. Then, as shown in Table 2, 47 patients discontinued the trial for chemotherapy unrelated reasons. Although it was considered in the protocol that 20% patients might not assessable for primary outcome, the censored patients number were relatively high, this might affect the accuracy of this trial. Furthermore, the evaluation criteria were based on the recommendations of PCWG1, [25] but some defects of these recommendations have been discovered recently. New response and progression criteria for PSA and symptoms have been proposed in the PCWG2, [19] e.g. Flare phenomena has been taken into consideration. Obviously, new criteria would make the results of clinical trial more reliable, however, adoption of conventional criteria was likely to make the current results comparable to previous outcomes.

## Conclusions

Compared with M3P arm, D3P arm significantly prolongs overall survival (8.21 months) and events PFS for patients with mCRPC. Although there is a higher incidence of grade 3–5 neutropenia with D3P arm than M3P arm, no other significant increasing of TEAEs incidence associated with D3P arm. Overall, this study suggests that D3P arm is safe, tolerable and effective for Chinese patients with mCRPC.

## Supporting Information

S1 Protocol Trial Protocol. (DOCX)

S1 CONSORT Checklist (DOC)

S1 Table Definition and criteria for efficacy assessments. (DOCX)

S2 Table Definition and criteria for hematologic toxicities. (DOC)
