# Supplementary material for: A Multicenter, Randomized Clinical Trial Comparing the Three-Weekly Docetaxel Regimen plus Prednisone versus Mitoxantone plus Prednisone for Chinese Patients with Metastatic Castration Refractory Prostate Cancer
Source: PLoS One. 2015 Jan 27;10(1):e0117002. doi: 10.1371/journal.pone.0117002 (PMC4307981; doi:10.1371/journal.pone.0117002)
Supplement: S1 Protocol — (DOCX) [file pone.0117002.s001.docx]

| **Study Protocol** | |
| --- | --- |
| **Current Primary Outcome Measures** | - Efficacy: Overall survival [ Time Frame: From beginning to end of the study ] - Objective response rate of patients with measurable disease by Response Evaluation Criteria in Solid tumours [ Time Frame: From beginning to end of study ] - Prostatic Specific Antigen response [ Time Frame: From the beginning to the end of study ] - Pain response (McGill-Melzack Scale) [ Time Frame: From beginning to end of study ] - Time to progression [ Time Frame: From beginning to end of study ] - Adverse event [ Time Frame: From beginning to end of study ] - Quality Of Life: Functional assessment of chronic illness therapy-prostate questionnaire will be used [ Time Frame: From beginning to end of study ] |
| **Original Primary Outcome Measures    (submitted: February 16, 2007)** | - Efficacy: Overall survival - Objective response rate by Response Evaluation Criteria in Solid tumours - Prostatic Specific Antigen response - Pain response (McGill-Melzack Scale) - Time to progression - Safety: incidence and severity of all adverse event - Quality Of Life: Functional assessment of chronic illness therapy-prostate questionnaire will be used |
| **Change History** | [Complete list of historical versions of study NCT00436839 on ClinicalTrials.gov Archive Site](https://clinicaltrials.gov/ct2/archive/NCT00436839) |
|  | |
| **Descriptive Information** | |
| **Brief Title** | Taxotere Prostate Cancer New Indication Registration Trial in China |
| **Official Title** | An Multicenter, Randomized Study of Comparison of Docetaxel Plus Prednisone With Mitoxantrone Plus Prednisone in the Patients With Hormone-refractory (Androgen-independent) Metastatic Prostate Cancer |
| **Brief Summary** | To compare overall survival after receiving mitoxantrone and prednisone or docetaxel and prednisone in subjects with hormone-refractory metastatic prostate cancer. |
| **Study Type** | Interventional |
| **Study Phase** | Phase 3 |
| **Study Design** | Allocation: Randomized Endpoint Classification: Safety/Efficacy Study Intervention Model: Parallel Assignment Masking: Open Label Primary Purpose: Treatment |
| **Condition** | Prostatic Neoplasms |
| **Intervention** | - Drug: Docetaxel   75mg/m² intravenously (day 1) every 21 days   - Drug: Mitoxantrone   12mg/m² intravenously every 21 days   - Drug: Prednisone   10mg orally given daily |
| **Study Arm (s)** | - Experimental: 1   Docetaxel 75mg/m² intravenously (day 1) every 21 days, plus prednisone 10mg orally given daily, minimal for 6 cycles and up to 10 cycle  Interventions:   - - Drug: Docetaxel   - Drug: Prednisone - Active Comparator: 2   Mitoxantrone 12mg/m² intravenously every 21 days, plus prednisone 10mg orally given daily, minimal for 6 cycles and up to 10 cycle  Interventions:   - - Drug: Mitoxantrone   - Drug: Prednisone |
|  | |
| **Recruitment Information** | |
| **Recruitment Status** | Completed |
| **Enrollment** | 228 |
| **Completion Date** | June 2012 |
| **Primary Completion Date** | June 2012   (final data collection date for primary outcome measure) |
| **Eligibility Criteria** | Inclusion Criteria:   - Histologically or cytologically proven prostate adenocarcinoma - Androgen independent prostate Cancer S/P orchiectomy and/or LHRH agonist Testosterone < 50 ng/dl (ie 1.735 nmol/l) - Documented progressive disease - Patients should have achieved stable analgesia for 7 days - Karnofsky Performance Status ≥ 70 - No prior treatment with cytotoxic agent (except estramustine) - Normal cardiac function must be confirmed by Left ventricular ejection fraction - Adequate organ function:   1. Hematology:      - Neutrophils > 1.5 x 10^9/L      - Hemoglobin > 10 g/dl. Erythropoietin use is allowed, but red blood cell transfusion to upgrade the hemoglobin level is not allowed      - Platelets > 100 x 10^9/L   2. Hepatic function:      - Total bilirubin < the upper-normal limit of the institution.      - Alanine aminotransferase and Aspartate transaminase < 1.5 times the upper-normal limit of the institution.   3. Renal function:      - Creatinine < 1.5 times the upper normal limit (ie National Cancer Institution grade < 1) - No brain or leptomeningeal metastases   Exclusion Criteria:   - Prior radiotherapy to >25% of bone marrow (whole pelvic irradiation is not allowed) - prior cytotoxic chemotherapy, except monotherapy with estramustine - prior isotope therapy - history of another cancer within the preceding five year - symptomatic peripheral neuropathy grade ≥ 2 - other serious illness or medical condition:   1. Congestive heart failure even if controlled. Previous history of myocardial infarction or angina pectoris within 1 year from study entry, uncontrolled hypertension or uncontrolled arrhythmias.   2. Active uncontrolled infection   3. Peptic ulcer, unstable diabetes mellitus or other contraindications for the use of corticosteroids.   4. Auto-immune disease (lupus, sclerodermia, rheumatoid polyarthritis) - treatment with any other anti-cancer therapy - treatment with bisphosphonates   The above information is not intended to contain all considerations relevant to a patient's potential participation in a clinical trial. |
| **Gender** | Male |
| **Accepts Healthy Volunteers** | No |
| **Location Countries** | China |
|  | |
| **Administrative Information** | |
| **NCT Number** | NCT00436839 |
| **Responsible Party** | Sanofi |
| **Study Sponsor** | Sanofi |
| **Investigators** | \| Study Director: \| Jing Fu \| Sanofi \|  \| \| --- \| --- \| --- \| --- \| |
| **Information Provided By** | Sanofi |
| **Start Date  ICMJE** | January 2007 |
| **Primary Completion Date** | June 2012   (final data collection date for primary outcome measure) |
| **Verification Date** | July 2012 |
| **Last Updated Date** | July 5, 2012 |
