# Supplementary material for: A Multicenter, Randomized Clinical Trial Comparing the Three-Weekly Docetaxel Regimen plus Prednisone versus Mitoxantone plus Prednisone for Chinese Patients with Metastatic Castration Refractory Prostate Cancer
Source: PLoS One. 2015 Jan 27;10(1):e0117002. doi: 10.1371/journal.pone.0117002 (PMC4307981; doi:10.1371/journal.pone.0117002)
Supplement: S1 Table — (DOCX) [file pone.0117002.s003.docx]

**Table S1 Definition and criteria for efficacy assessments**

| Definition and Criteria for Efficacy Assessments | |
| --- | --- |
| **Assessments** | **Criteria** |
| Overall Survival | Defined as the time between randomization and the date of death (whatever the cause), patients who were still alive at their last contact or at the cut-off date of the analysis were censored at their date of last contact for the OS analysis. |
| Progression Free Survival | Progression free survival for pain, PSA, tumor and disease was calculated between the date of randomization to the date of event progression or death (due to prostate cancer or study drug), whichever occurred first. |
| Durations of Response | Durations of response for pain, PSA, tumor were calculated from the date response was achieved to the date on which disease progression was first observed |
| Pain Response | Pain response, which applied only to patients with median PPI>2 on McGill-Melzack scale, was defined as a 2-point or greater reduction with no increase in analgesic score,or a reduction of at least 50% in analgesic use from basline analgesic score with on increase in pain |
| Pain Progression | Defined as an increased of ≥1 point in the PPI scale from its nadir noted on two consecutive visits three weeks apart, or ≥25% increase in the daily analgesics score compared with the baseline score and noted on two consecutive visits three weeks apart, or requirement for local palliative radiotherapy |
| PSA Response | Defined as a PSA decline of *>*50% and confirmed at least three weeks later |
| PSA Progression | - In PSA non-responders: progression was defined as a 25% increase over the nadir value (provided that the rise is a minimum of 5ng/ml) and confirmed by a second value at least one week later.  - In PSA responders: progression was defined as a 50% increase over the nadir value (provided that the rise is a minimum of 5ng/ml) and confirmed by a second value at least one week later. |
| Tumor assessment | complete response = disappearance of all target lesions  partial response = 30% decrease in the sum of the longest diameter of target lesions  progressive disease= 20% increase in the sum of the longest diameter of target lesions  stable disease= small changes that do not meet above criteria |
| Quality of Life | The major instrument for quality of life evaluation is Functional Assessment of Cancer Therapy-Prostate (FACT-P) questionnaire, version 4. |
| Quality of Life Response | Quality of life response for a patient will be considered as a 16 point improvement in FACT-P score for 2 consecutive visits as compared to baseline |
| PSA= prostate specific antigen; PPI= Present Pain Intensity | |
