# Supplementary material for: A Multicenter, Randomized Clinical Trial Comparing the Three-Weekly Docetaxel Regimen plus Prednisone versus Mitoxantone plus Prednisone for Chinese Patients with Metastatic Castration Refractory Prostate Cancer
Source: PLoS One. 2015 Jan 27;10(1):e0117002. doi: 10.1371/journal.pone.0117002 (PMC4307981; doi:10.1371/journal.pone.0117002)
Supplement: S2 Table — (DOC) [file pone.0117002.s004.doc]

Table S2 Definition and Criteria for hematologic toxicities.
Common Toxicity Criteria of the National Cancer Institute for hematologic toxicities(version 3.0)	
		Grade 		
Adverse Event 	Short Name 	1 	2 	3 	4 	5 	
Bone marrow cellularity 	Bone marrow cellularity 	Mildly hypocellular 
or ¡Ü25% reduction from normal cellularity for age 	Moderately hypocellular 
or >25 – ¡Ü50% reduction from normal cellularity for age 	Severely hypocellular 
or >50 – ¡Ü75% reduction cellularity from normal for age 	— 	Death 	
Haptoglobin 	Haptoglobin 	<LLN 	— 	Absent 	— 	Death 	
Hemoglobin 	Anemia	<LLN – 10.0 g/dL  
<LLN – 6.2 mmol/L 
<LLN – 100 g/L 	<10.0 – 8.0 g/dL
<6.2 – 4.9 mmol/L  
<100 – 80g/L 	<8.0 – 6.5 g/dL
<4.9 – 4.0 mmol/L 
<80 – 65 g/L 	<6.5 g/dL
<4.0 mmol/L
<65 g/L 	Death 	
Hemolysis (e.g., immune hemolytic anemia, drug-related hemolysis) 	Hemolysis 	Laboratory evidence of hemolysis only (e.g., direct antiglobulin test [DAT, Coombs'] schistocytes) 	Evidence of red cell destruction and ¡Ý2 gm decrease in hemoglobin, no transfusion 	Transfusion or medical intervention (e.g., steroids) indicated 	Catastrophic consequences of hemolysis (e.g., renal failure, hypotension, bronchospasm, emergency splenectomy) 	Death 	
Leukocytes (total WBC) 	Leukopenia	<LLN – 3000/mm3 
<LLN – 3.0 x 10^9^ /L 	<3000 – 2000/mm3 
<3.0 – 2.0 x 10^9^ /L 	<2000 – 1000/mm3 
<2.0 – 1.0 x 10^9^ /L 	<1000/mm3
<1.0 x 10^9^ /L 	Death 	
Lymphopenia  	Lymphopenia 	<LLN – 800/mm3 
<LLN x 0.8 –109 /L 	<800 – 500/mm3 
<0.8 – 0.5 x 10^9^ /L 	<500 – 200 mm3
 <0.5 – 0.2 x 10^9^ /L 	<200/mm3
<0.2 x 10^9^ /L 	Death 	
Myelodysplasia 	Myelodysplasia 	— 	— 	Abnormal marrow cytogenetics (marrow blasts ¡Ü5%)  	RAEB or RAEB-T (marrow blasts >5%) 	Death 	
Neutrophils/granulocytes (ANC/AGC) 	Neutropenia 	<LLN – 1500/mm3
<LLN – 1.5 x 10^9^ /L 	<1500 – 1000/mm3 
<1.5 – 1.0 x 10^9^ /L 	<1000 – 500/mm3 
<1.0 – 0.5 x 10^9^/L 	<500/mm3<0.5 x 10^9^ /L 	Death 	
Platelets 	Platelets 	<LLN – 75,000/mm3
<LLN – 75.0 x 109 /L 	<75,000 – 50,000/mm3 <75.0 – 50.0 x 109 /L 	<50,000 – 25,000/mm3 <50.0 – 25.0 x 109 /L 	<25,000/mm3 
<25.0 x 109 /L 	Death 	
Splenic function 	Splenic function 	Incidental findings (e.g., Howell-Jolly bodies) 	Prophylactic antibiotics indicated 	— 	Life-threatening consequences 	Death 	
LLN=lower limits of normal	
